# The impact of tympanic membrane perforations on middle ear transfer function

**DOI:** 10.1007/s00405-021-07078-9

**Published:** 2021-09-27

**Authors:** Nicholas Bevis, Benjamin Sackmann, Thomas Effertz, Michael Lauxmann, Dirk Beutner

**Affiliations:** 1 Department of Otolaryngology, University of Goettingen, Robert-Koch-Straße 40, 37075 Göttingen, Germany; 2 Department of Mechanical Engineering, University of Reutlingen, 72762 Reutlingen, Germany

**Keywords:** Tympanic membrane, Tympanic membrane perforations, Middle ear transfer function, Finite element method, FEM, Laser-Doppler vibrometry, Middle ear mechanics, Tympanoplasty

## Abstract

**Purpose:**

Injury or inflammation of the middle ear often results in the persistent tympanic membrane (TM) perforations, leading to conductive hearing loss (HL). However, in some cases the magnitude of HL exceeds that attributable by the TM perforation alone. The aim of the study is to better understand the effects of location and size of TM perforations on the sound transmission properties of the middle ear.

**Methods:**

The middle ear transfer functions (METF) of six human temporal bones (TB) were compared before and after perforating the TM at different locations (anterior or posterior lower quadrant) and to different degrees (1 mm, ¼ of the TM, ½ of the TM, and full ablation). The sound-induced velocity of the stapes footplate was measured using single-point laser-Doppler-vibrometry (LDV). The METF were correlated with a Finite Element (FE) model of the middle ear, in which similar alterations were simulated.

**Results:**

The measured and calculated METF showed frequency and perforation size dependent losses at all perforation locations. Starting at low frequencies, the loss expanded to higher frequencies with increased perforation size. In direct comparison, posterior TM perforations affected the transmission properties to a larger degree than anterior perforations. The asymmetry of the TM causes the malleus-incus complex to rotate and results in larger deflections in the posterior TM quadrants than in the anterior TM quadrants. Simulations in the FE model with a sealed cavity show that small perforations lead to a decrease in TM rigidity and thus to an increase in oscillation amplitude of the TM mainly above 1 kHz.

**Conclusion:**

Size and location of TM perforations have a characteristic influence on the METF. The correlation of the experimental LDV measurements with an FE model contributes to a better understanding of the pathologic mechanisms of middle-ear diseases. If small perforations with significant HL are observed in daily clinical practice, additional middle ear pathologies should be considered. Further investigations on the loss of TM pretension due to perforations may be informative.

**Supplementary Information:**

The online version contains supplementary material available at 10.1007/s00405-021-07078-9.

## Introduction

Perforations of the tympanic membrane (TM) can be the result of acute and chronic middle-ear disease or after trauma. In addition, invasive procedures such as intratympanic injection, transtympanic electrical stimulation of the cochlea or the frequently performed tympanic tube insertion also lead to a perforation of the TM [1], but not necessarily to conductive hearing loss (HL). Although the TM has a high spontaneous healing tendency [2], long-term perforations may occur. These perforations can lead to conductive HL and recurrent middle-ear infections [3].

In addition to solitary TM perforations, other pathologies are frequently discovered during surgery. These include tympanosclerosis, malleus ligament fixation, and discontinuity of the incudostapedial joint [4]. The extent to which a perforation leads to HL is highly variable and subject of research [5]. A tympanostomy tube with at least 1 mm internal diameter or other small perforations usually result in a miniscule conductive HL, especially in lower frequencies [6]. If there is a discrepancy between clinical findings and HL, further pathology in the middle ear must be assumed [7].

The general belief that the location of a TM perforation has an impact on HL is widespread but often disputed. Clinicians generally assume that perforations of the posterior quadrants of the TM cause greater HL than perforations of the anterior quadrants [3], although this view has been refuted by several studies [7–10]. Notably, the underlying mechanism of hearing loss is unclear and the pathologic mechanisms of middle ear disease are not fully understood [11].

This study aims to investigate how the size and location of a TM perforation influence the middle ear transfer function (METF), which is the ratio between the stapes piston-like displacement and the acoustic ear canal sound pressure. We also used a preexisting Finite Element (FE)-model of the middle ear [12–14] to test TM perforation effects. Analysis of the movement of individual middle ear structures in the FE-model allowed us to test causal relationships between middle ear pathology and altered ossicular movements.

Correlation of the experimental measurements in the temporal bones (TB) with theoretical FE simulations provides insight into the pathological mechanisms of middle ear disease.

## Methods

### Temporal bone preparation

**Fig. 1:**
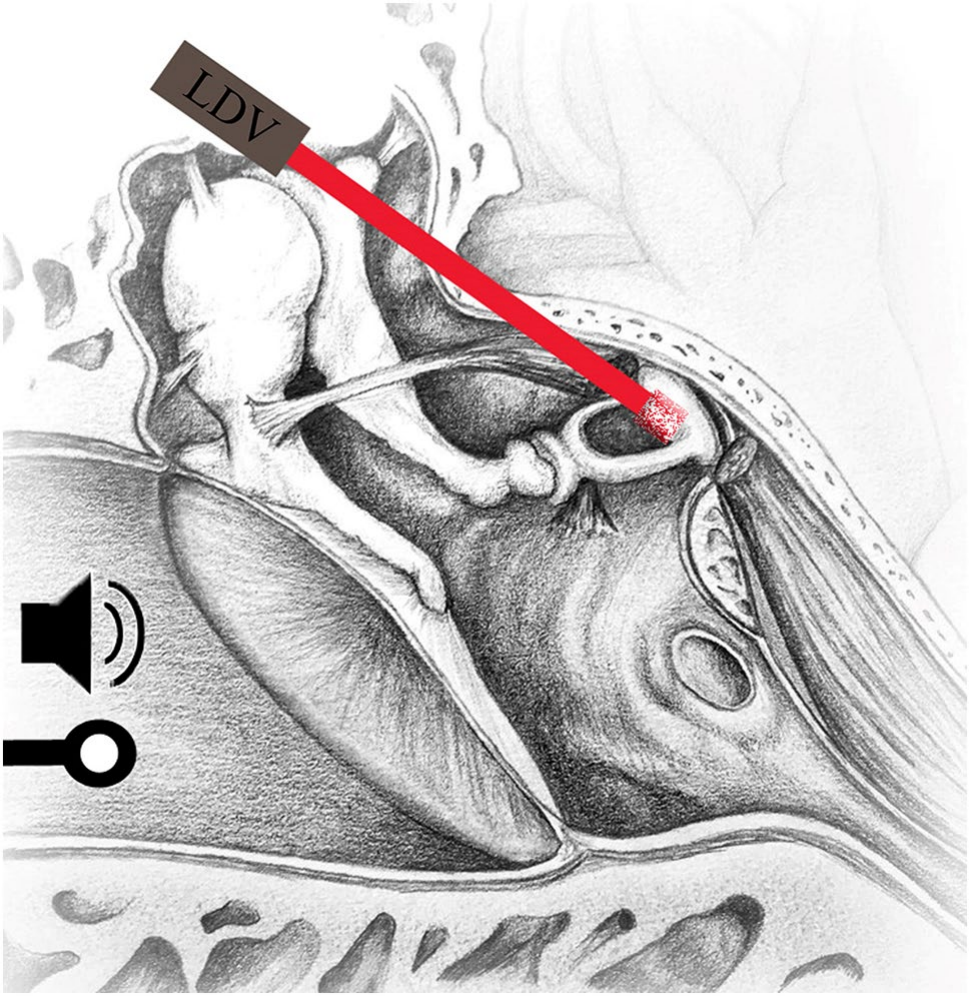
Experimental setup for the stapes footplate velocity measurements with LDV. The laser is pointed at a reflector foil on the stapes footplate. Sound stimulation through the ear canal evoke vibrations of the TM, which in turn are transferred to the stapes by the ossicular chain. A microphone in the ear canal is used as a pressure reference for the calculated METF

**Fig. 2:**
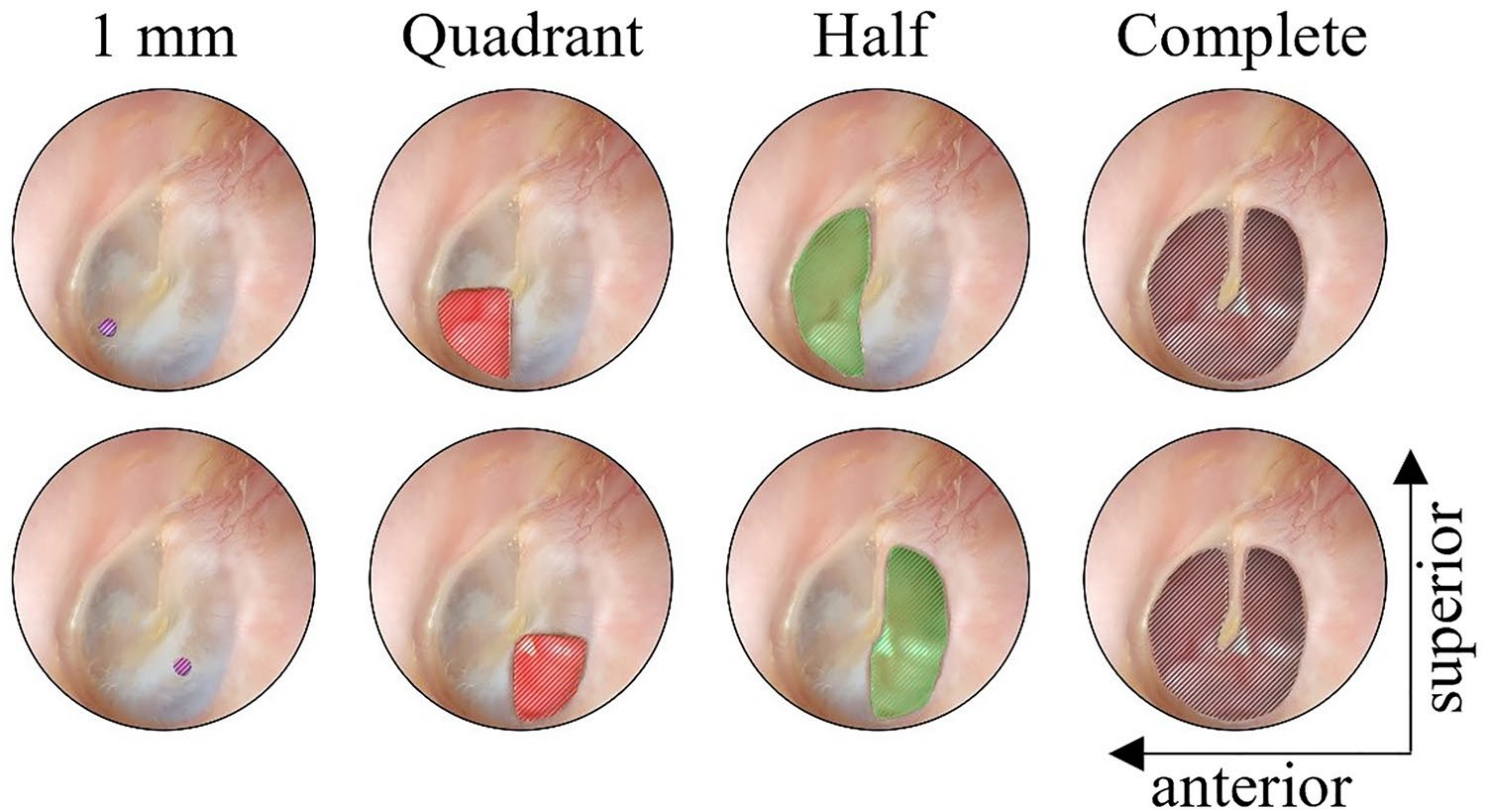
Location of TM perforations increasing in size from left to right (1 mm; ¼; ½; complete perforation). Upper pictures depict the perforations of the anterior quadrant, lower pictures of the posterior quadrant. The color-coding correlates to Figs. 4 and 5, as well as supplementary Fig. 1

Six TBs were frozen postmortem according to the American Society for Testing and Materials (ASTM F2504-05) recommendation [15, 16] without preservatives and thawed right before measurements. First, access to the ossicular chain and the stapes footplate was created via a facial recess approach. The tympanic cavity was therefore partly open. Next, a reflector foil was placed on the stapes footplate (Fig. 1). The first measurements were taken with an intact TM, followed by measurements after ever-increasing perforation size (1 mm > ¼ > ½ > complete perforation) in either the anterior or posterior quadrants (Fig. 2). Out of the six TBs, three were used for the anterior perforations and three for the posterior perforations. A sickle knife was used to perforate the TM. The uniformity of the 1 mm perforations was achieved by using a surgical suction device with 1 mm diameter as a template. The annulus fibrosus was respected in all preparations and the TBs were kept moist by applying the saline solution. This prevented drying and stiffening [17]. One temporal bone at a time was used for either anterior or posterior perforation measurements to ensure experimental conditions, shortly after thawing.

### Experimental setup

The measurements were performed on a CleanBench™ vibration-isolation table (TMC, Peabody, Massachusetts, USA). To achieve a hermatically sealed outer ear canal, the specimens were fitted with a closed earpiece in the bony part of the outer ear canal in 5 mm distance of the TM and fixed with two-component adhesive. A silicone tube carrying a sound source (ER-2, Etymotic Research, Elk Grove Village, Illinois, USA) and a microphone (ER-7C, Etymotic Research, Elk Grove Village, Illinois, USA) were included in the closed earpiece. A laser doppler vibrometer (LDV) (CLV 1000, Polytec GmbH, Waldbronn, Germany) attached to a surgical microscope with a micromanipulator-controlled prism was pointed at a reflector foil positioned on the stapes footplate between the posterior and anterior crus. The acceleration of the center of the stapes footplate was measured with the LDV through the facial recess during sound excitation. Using an exponential chirp, a stimulus between 100 and 10,000 Hz was generated.

Data were acquired using Polytec Software (VibSoft Data Acquisition Software) and displayed in a logarithmic scale. Results were analyzed with Origin Pro 8.2 (OriginLab Corporation, Northampton, USA). Graphs were presented as mean values or relatives to measurements. Unless otherwise indicated, analysis was done on six individual specimen. Data were analyzed by unpaired *t* test, after testing for normal distribution with a Shapiro–Wilk test. It was found that over 95% of the measured data were normally distributed for anterior perforations and over 93% of the data were normally distributed for posterior perforations. A *p* value of at least < 0.05 was considered as significant.

### Finite element model

**Fig. 3:**
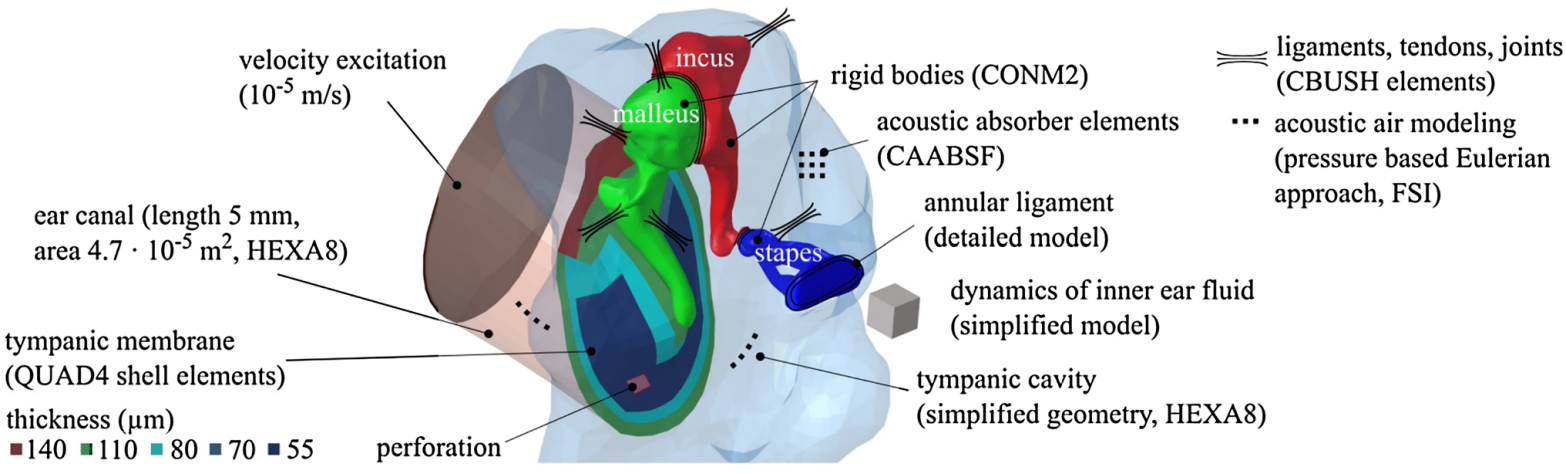
FE model of the middle ear used for the simulation of TM perforations. The model has an acoustic ear canal, a tympanic cavity, an elastic TM, and an ossicular chain

The FE model of the middle ear used in this study is implemented in Hyperworks (Altair Engineering, Inc., Troy, MI) and is shown in Fig. 3. The geometry of the ear canal is reconstructed from micro-CT data. The geometry of the tympanic cavity is simplified by using plane side walls. The volume of the cavity is 9 × 10^–7^ m^3^ according to TB 24L in [18], which was used to validate the FE model [14]. The aditus ad antrum acts as a bottleneck connecting the air volume in the mastoid cells and the tympanic cavity. Since the main functions of the mastoid cells are gas exchange and static pressure compensation, the enclosed air volume of 9 × 10^–7^ m^3^ is roughly modelled as one mass oscillator. This corresponds to a simplified model of Helmholtz resonance phenomena of air oscillating between two cavities. The air in the ear canal and tympanic cavity is modelled with hexahedral acoustic finite elements (HEXA8, MAT10). Acoustic absorber elements (CAABSF) take into account the opening in the tympanic cavity due to the posterior tympanotomy. The absorber elements model a Sommerfeld boundary condition of 2.5 mm diameter. The fluid-structural coupling between the TM and the adjacent air is implemented using a pressure-based Eulerian approach. The ear canal wall is considered rigid by fixing all degrees of freedom.

The geometry of the TM is reconstructed from micro-CT data as well. It is meshed using first-order shell elements (QUAD4, MAT1) and fully clamped at its boundary. For the TM, five regions with constant thickness are defined, as shown in Fig. 3, which were derived from the characteristic relative thickness distribution measured in Kuypers et al. [19]. The perforations of the TM introduced in the experiment are considered in the FE model by a corresponding hole in the FE shell mesh of the TM. The two-fluid compartments in the ear canal and the tympanic cavity are thus directly connected via the perforation at the location of the perforation and no longer via the fluid–structure interaction.

The ossicles are modelled as rigid bodies (CONM2) and characterized by their mass and inertia, because the ossicles deformation can be neglected up to 10 kHz. Whereas malleus and incus have all 6 degrees of freedom, the stapes is further constrained, allowing only a translational piston motion along the y-axis and two rotational (rocking) motions around the x- and z-axis of the stapes coordinate system. Regarding the stapes footplate motion measured in the LDV measurements, this means that the bearing of the stapes in the simulation model also does not allow any other form of motion except the piston-like motion. The ligaments, tendons and joints are represented by passive spatial spring-damper elements. The annular ligament is modelled according to measurements from Lauxmann et al. [20], who derived the stiffness characteristics of the annular ligament from point-stiffness measurements on human TBs. Eight translational and two rotational progressive nonlinear springs, distributed along the circumference of the stapes footplate, modelled the derived inhomogeneous stiffness distribution of the annular ligament.

The model used here is based on Lauxmann [21] and uses the same parameters for the ossicles’ masses and moments of inertia, ligament stiffnesses and damping parameters. The model in Lauxmann [21] was validated on a measured individual METF measured on the stapes. In Sackmann et al. [13], the model has been shown to simulate wideband tympanometry appropriately and the simulations of energy reflectance were compared to published measurements and simulation results. The model was further validated on impedance measurements from Voss et al. [18] and compared to their published mean and standard deviation of normal TBs and an individual TB with removed TM, both with a sealed tympanic cavity. To match the TB measurements and the experimental conditions of Voss et al. [18] to the model from Lauxmann [21], the Youngs modulus of the pars tensa in Sackmann et al. [14] was reduced from 4 MPa to 3.2 MPa, the ear canal length was shortened to the experimental conditions of the reference, the local TM thickness values were adapted slightly as shown in Fig. 3 as well as Sackmann et al. [14] and the size and boundary conditions of the tympanic cavity were changed [14]. The METF of the model in Sackmann et al. [14] was assured to stay within the standard throughout all these modifications for the here investigated frequency range 0.2–4 kHz. Therefore, the effect on METF should be reproduced appropriately in this study and acoustics of the ear canal and cavity should also be reproduced appropriately, as this model reproduces the effects of intact and removed tympanic membranes well as showed in Sackmann et al. [14].

Compared to Sackmann et al. [14], the only changes to the here investigated model, are the acoustic opening of the tympanic cavity using CAABSF-Elements and the perforations of the TM of several sizes and locations.

## Results

### Effect of TM perforation size on the middle ear transfer function

**Fig. 4:**
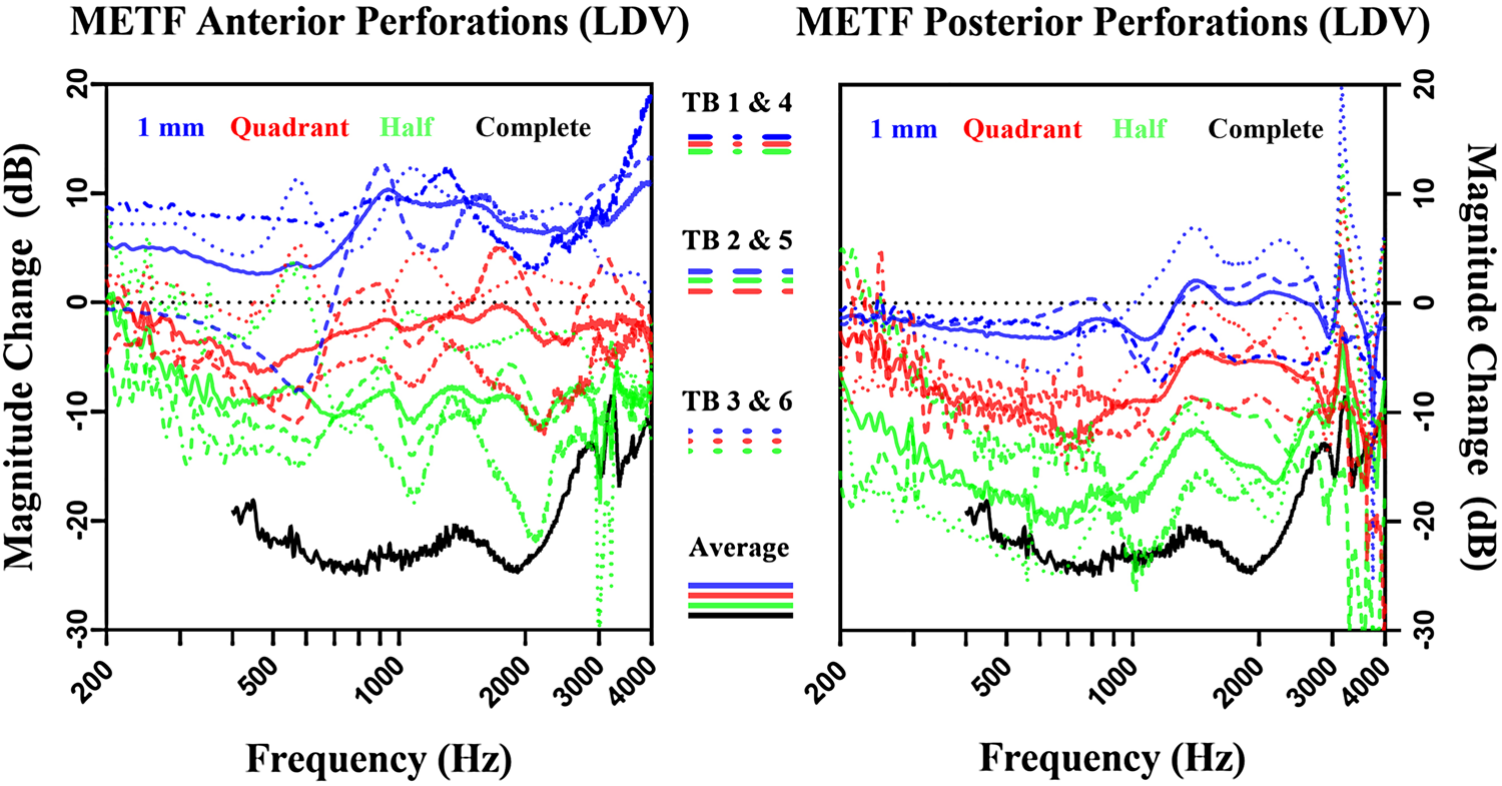
Magnitude of change in dB of METF (as ratio between the stapes piston-like displacement and the acoustic ear canal sound pressure) of anterior perforations (left) and posterior perforations (right) in relation to the intact TM. The dotted lines represent individual TB measurements, whereas the solid lines represent the mean of all TB (color-coding as depicted in Fig. 2). The data of the complete perforation (black) of the TM is not shown below 400 Hz due to the high noise level (because of significantly reduced absolute velocities)

The amplitude of stapes footplate velocities during acoustic stimulation showed frequency and perforation size-dependent losses in all six TBs. Starting at low frequencies, the loss of sound transmission expands to higher frequencies with increased perforation size. The larger the perforation, the higher the noise level at lower frequencies (due to the significantly reduced absolute velocities). The magnitude change of individual TB is shown in Fig. 4, as well as an average of all TBs with the same perforation size and location. The variation between TBs when comparing perforations of the same size is high. The perforation covering the entire TM results into a relative decrease of up to 27 dB compared to the intact TM (Fig. 4).

### Effect of TM perforation location on the middle ear transfer function

Anterior TM perforations show less profound effects on the METF than posterior perforations. When comparing 1 mm perforations, significant differences (*p* < 0.05, 95% confidence interval) between the anterior and posterior group can be observed in the frequency range of 703–1208, 2747–3041 and 3751–3786 Hz. With increasing perforation size and thus loss of lower frequencies, the significantly different frequency range decreases (Quadrant: 643–952, 2795–2895 & 3425–3597 Hz; Half: 758–774 & 802–914 Hz). At 900 Hz, the average magnitude change between anterior/posterior is as follows: 1 mm 9.95/− 1.71 dB (*p* = 0.012); Quadrant − 1.65/− 9.48 dB (*p* = 0.040); Half − 8.30/− 17.85 dB (*p* = 0.047) (see Fig. 4). Comparing complete perforations between the TBs used for anterior and posterior perforations, no significant difference in the measured frequency range can be noted. A complete perforation leads to an average magnitude change of 22.40 dB relative to controls.

### Finite element model

**Fig. 5:**
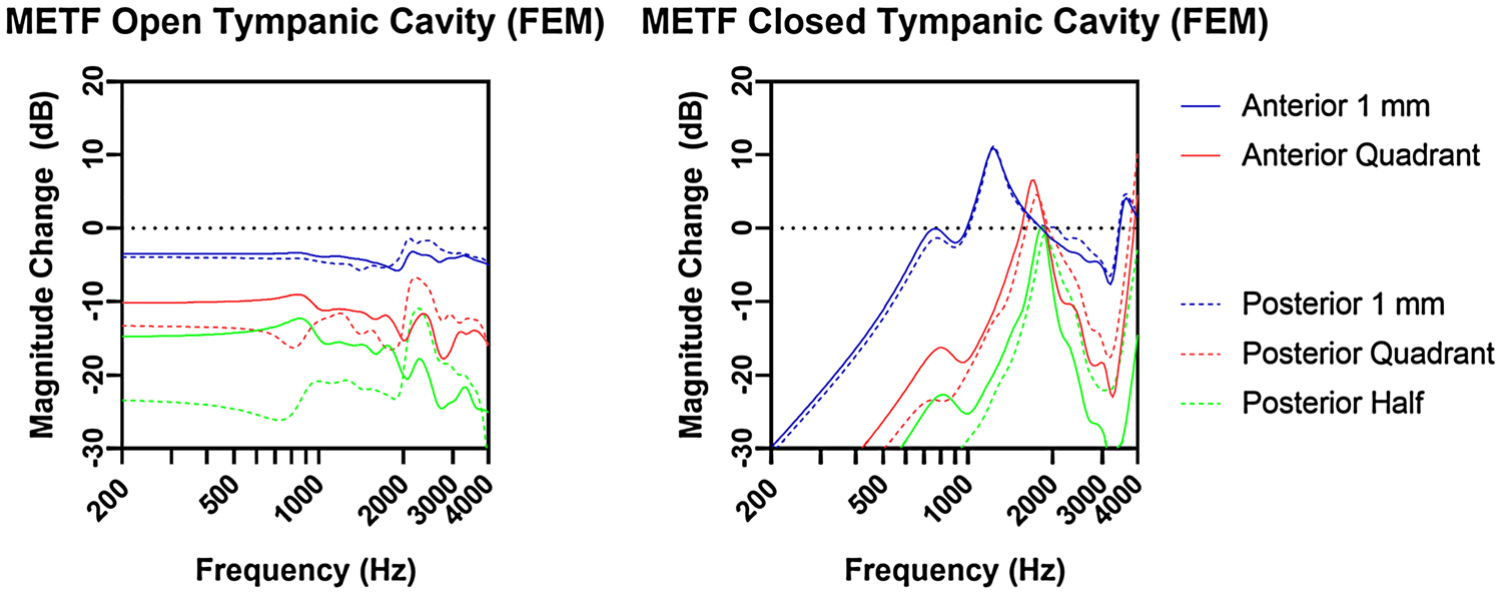
Magnitude of change in dB of METF in relation to the intact TM for an open mastoid/tympanic cavity (left) and acoustically sealed tympanic cavity (right) for anterior (solid lines) and posterior (dashed lines) TM perforations of different sizes calculated using individual TB simulations in the FE model. The color-coding correlates to Fig. 2

By means of FE simulation, we confirmed our findings that anterior TM perforations have a less profound effect on the METF than posterior perforations at frequencies below 2000 Hz. The magnitude changes increase for larger perforation sizes (Fig. 5). The leak-tightness of the tympanic cavity significantly influences the perforation-induced losses at low frequencies. For an acoustically sealed tympanic cavity, and a tympanic cavity with a very small opening (1 mm and quadrant perforation), we observed an increase in the motion amplitude of the TM and stapes footplate velocity by perforation, mainly above 1000 Hz (Fig. 5, right).

## Discussion

Our findings support the widespread opinion, that posterior perforation of the TM result in larger conductive HL [3]. Significant differences were found in the assessment of equally sized perforations of the lower anterior and lower posterior quadrant in the mentioned frequency ranges (Fig. 4). As the perforation size increases, the frequency range showing a significant difference becomes smaller. Starting with small TM perforations, we observed low frequency-METF amplitude losses, which expanded to higher frequencies with increased perforation size. In addition, a 1 mm perforation of the lower anterior quadrant resulted in an increased METF amplitude relative to an intact TM.

FE model simulations with a sealed tympanic cavity show that small perforations lead to a decrease in TM rigidity and thus to an increase in oscillation amplitude of the TM, possibly explaining the larger METF amplitudes observed in 1 mm perforations of the lower anterior quadrant. In contrast, the METF amplitude decreases more in posterior perforations. One explanation why more HL is observed in posterior perforations might be related to the asymmetry of the TM, whereby the deflection amplitudes are larger in the posterior TM quadrants than in the anterior quadrants. Which in turn suggests that perforations in the posterior quadrants should have a stronger effect than perforations in the anterior quadrants (Fig. 5). The FE model simulations show that the open tympanic cavity measurements are different when compared to the closed tympanic cavity measurements. However, this has no consequences in terms of the clinical significance of the LDV measurements when comparing relative measurements between anterior and posterior perforations. Xie et al. [11] correlated clinical findings on blast-induced TM perforations with their FE model simulation of the middle ear showing a frequency-dependent effect of perforation location on the METF amplitude. Interestingly, the effects on the METF did not rely on perforation location for small perforation sizes.

Comparing the magnitude change from posterior perforations to anterior perforations (see supplementary Fig. 1), the LDV measurements and FE model simulations show similar characteristics for METF amplitude loss up to 2 kHz, except for the perforation with a size of 1 mm. These qualitative differences between simulation and measurement for the small 1 mm perforation could be explained by the pretension of the TM, which is not considered in the FE simulation. The perforation reduces the initial pretension in the TB experiment. According to Caminos [9, 22], who investigated the influence of a homogenous change in pretension, increasing stapes displacements in the low-frequency range can be expected. However, a perforation leads to an inhomogeneous decrease of pretension of the TM, which makes a prediction from simulations difficult. Further experimental investigations on the loss of pretension of a perforated TM are necessary. In the FE model, perforations of the anterior quadrants show larger METF amplitudes than perforations of the posterior quadrants below 2000 Hz, as shown in supplementary Fig. 1. At higher frequencies, the opposite is true. However, we did not observe this effect in our LDV measurements. Considering the FE simulation results shown in Fig. 5 and the measurements from Voss and Rosowski [9], it is likely that with increasing perforation size resonances in the frequency range of 1–2 kHz shift to higher frequencies. Depending on the position of the perforation, the magnitude of the shift is slightly different. The reduction of the pretension counteracts the transmission loss through the perforation regarding the stapes displacements. Since the transmission loss is more prominent on perforations of the posterior quadrants, as shown in both the experiment and the simulation, the increase in stapes displacement magnitude due to the reduction of the pretension is barely if at all observed on perforations of the posterior quadrants.

Voss et al. [7] described that loss in middle ear sound transmission depends on frequency, perforation size and middle ear volume. However, they showed no dependence of sound transmission on the location of a TM perforation. In contrast, other groups were able to determine the dependence of middle ear sound transmission on the location of a perforation in their experiments [23, 24] and its effect on HL [25, 26]. Voss et al. [27] demonstrated that higher sound pressure at the oval window has no relevant effect on sound transmission. The herein-mentioned “acoustic route” describes the effect of direct sound pressure stimulation of the oval and round window in perforated TM. The contribution is generally negligible since hearing mainly results from a reduction in sound coupling rather than direct sound pressure at the oval or round window. The “acoustic route” has a relevant effect on sound transmission at lower frequencies in TM with larger perforations [9]. The FE model does not account for the contribution of the acoustic route. Furthermore, there are unpredictable effects that are difficult to simulate, e.g. remaining loose parts of the TM leading to acoustic artifacts.

Perforations in our study of fresh-frozen TBs correlate with acute injuries, whereas many clinical findings reflect conditions in which other middle ear pathologies may be present [7–9]. Understanding the characteristics of TM perforations and their contribution to HL may help identify additional middle-ear diseases not expected on clinical examination. In addition, a more detailed knowledge of the transmission properties of the TM can help in counseling patients regarding their prognostic hearing level and the contribution of perforations to their hearing. If the surgeon chooses stiffer materials such as cartilage and thicker slices for more stability in the reconstruction of the TM, it will affect the oscillation amplitude of the TM and thus the hearing level.

In a clinical approach, Mehta et al. [10] compared posterior and anterior perforations, showing no significant correlation between location and HL, although a trend for a larger air–bone gap of posterior perforations compared to anterior perforations was observed in all frequencies similar to the results of Virk and colleagues [28]. The anatomy and mechanics of the middle ear varies considerably across ears, consequently perforations also effect sound transmission differently. Lerut et al. [5] found a significant correlation of perforation location in perforations with umbo involvement in his prospective clinical trial of 220 patients. Lerut et al. described a smaller influence of perforations on the resonance frequency of 2 kHz, which we also found in our LDV-measurements.

While Voss et al. [9] show a decrease of 40 dB/decade in the quasi-static range, our measurements (Fig. 4) and simulations with an open tympanic cavity (Fig. 5, left), which corresponds to our measurements conditions, show a rather flat course for low frequencies. Simulations of the FE model suggest that these differences are mainly due to differences in the preparations. In Voss et al. [9] the tympanic cavity is acoustically sealed, whereas in this study it is opened due to the facial recess approach. By modelling a sealed tympanic cavity, the losses in the quasi-static range are significantly lower and the simulation results (Fig. 5, right) are comparable with Voss et al. [9]. The excitation amplitude of the TM is determined by the pressure difference between the ear canal and the tympanic cavity. In contrast to the intact TM, the perforation of the TM leads to a partial pressure equalization on both sides of the TM resulting in a strong decrease in the excitation amplitude of the TM at low-frequency excitation. Another opening in the tympanic cavity may lead to phase differences in the pressure between the tympanic cavity and the auditory canal, which in turn excites the TM more and reduces the amplitude drop during low-frequency excitation.

Up to date, this study is unique to describe effects of specific perforations on the METF. The methodical approach was lacking reproducibility in perforations bigger than 1 mm, since they were manually applied using a surgical sickle knife. The measured bones show a larger spread in variance in higher perforation sizes (Fig. 4). The FE-model was adapted to fit the anatomical measuring conditions of the experimental setup. By means of the FE model, important cause-effect relationships can be determined, which are sometimes very difficult to investigate in measurements, such as the influence of the tympanic cavity preparation in this study. However, to better map the complex mechanisms of middle ear perforations with an FE model, much more consistent and comprehensive measurement data from individual ears were needed.

In summary, the measured velocities of the stapes footplate after acoustic excitation showed frequency and size-dependent losses in both perforation locations. Even with small perforations, a loss of low frequencies was found, which spread to higher frequencies as the perforation size increased. In direct comparison of location, posterior TM perforations impair the transmission properties more than anterior perforations. If small perforations with significant HL are observed in daily clinical practice, additional middle ear pathologies must be considered.

## Conclusions

Size and location of TM perforations have a characteristic influence on the METF. The correlation of the experimental LDV measurements with an FE model contributes to a better understanding of the pathologic mechanisms of middle-ear diseases. If small perforations with significant HL are observed in daily clinical practice, additional middle ear pathologies should be considered. Further investigations on the loss of TM pretension due to perforations may be informative.

## Supplementary Information

Below is the link to the electronic supplementary material.

Supplementary Fig. 1: Difference in Magnitude of anterior perforations compared to posterior perforations (posterior–anterior). METF of the mean of the LDV measurements (solid lines) and the FE model calculation (dashed lines). FE model based on an open tympanic cavity. The color-coding correlates to Fig. 2. (TIF 300 KB)
