# Supplementary figures and images for: The impact of tympanic membrane perforations on middle ear transfer function
Source: Eur Arch Otorhinolaryngol. 2021 Sep 27;279(7):3399–406. doi: 10.1007/s00405-021-07078-9 (PMC9130167; doi:10.1007/s00405-021-07078-9)

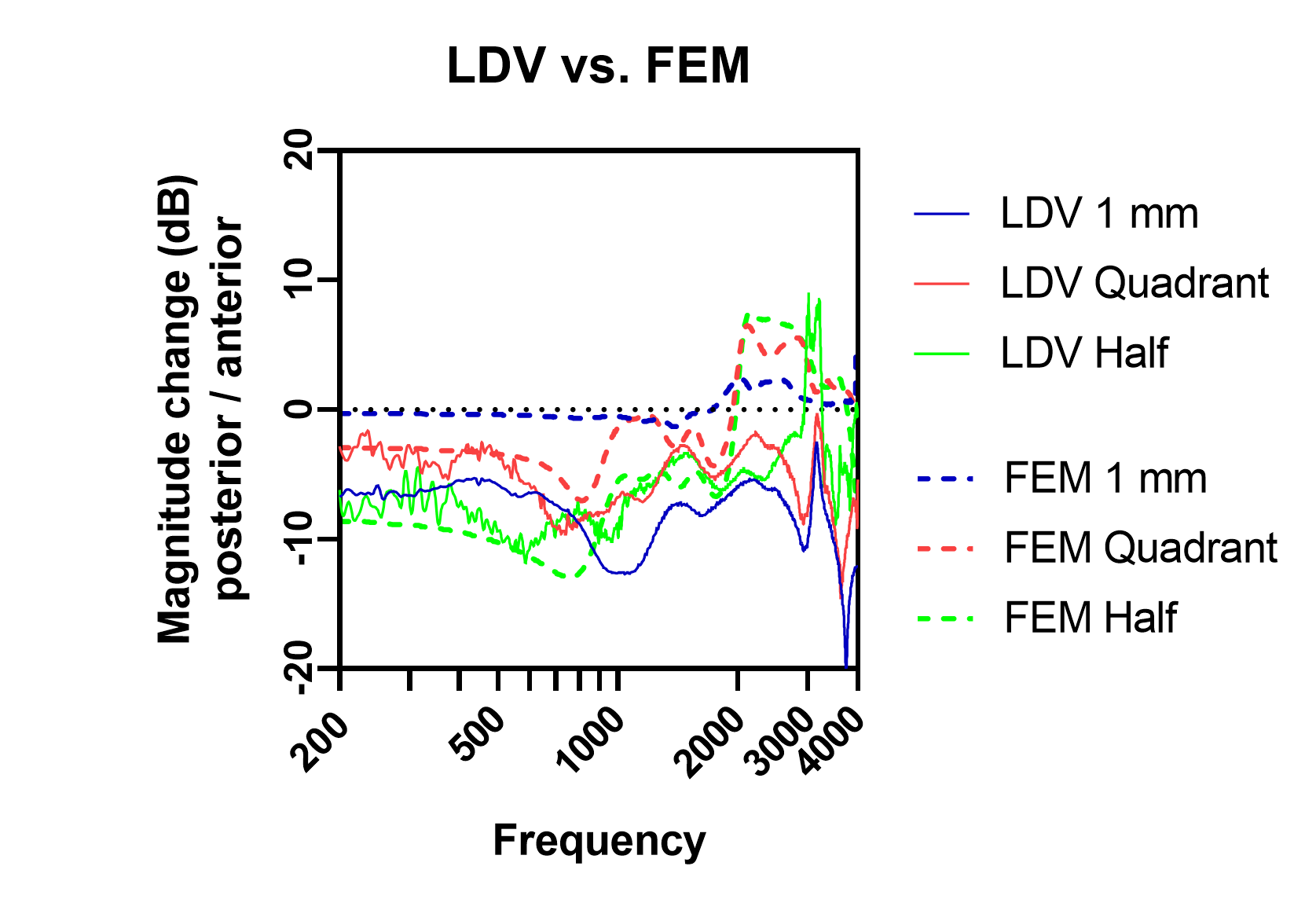

Supplement: Supplementary file 1 — Supplementary Fig. 1: Difference in Magnitude of anterior perforations compared to posterior perforations (posterior–anterior). METF of the mean of the LDV measurements (solid lines) and the FE model calculation (dashed lines). FE model based on an open tympanic cavity. The color-coding correlates to Fig. 2. (TIF 300 KB) [file 405_2021_7078_MOESM1_ESM.tif]
